# Immunological and hematological outcomes following protracted low dose/low dose rate ionizing radiation and simulated microgravity

**DOI:** 10.1038/s41598-021-90439-5

**Published:** 2021-06-01

**Authors:** Amber M. Paul, Eliah G. Overbey, Willian A. da Silveira, Nathaniel Szewczyk, Nina C. Nishiyama, Michael J. Pecaut, Sulekha Anand, Jonathan M. Galazka, Xiao Wen Mao

**Affiliations:** 1 Space Biosciences Division, NASA Ames Research Center, Moffett Field, CA 94035 USA; 2 Universities Space Research Association, Columbia, MD 21046 USA; 3 Department of Human Factors and Behavioral Neurobiology, Embry-Riddle Aeronautical University, Daytona Beach, FL 32114 USA; 4 Department of Genome Sciences, University of Washington, Seattle, WA 98195 USA; 5 Faculty of Medicine, Health and Life Sciences, School of Biological Sciences, Institute for Global Food Security (IGFS), Queen’s University, Belfast, BT9 5DL Northern Ireland UK; 6 Ohio Musculoskeletal and Neurological Institute and Department of Biomedical Sciences, Heritage College of Osteopathic Medicine, Ohio University, Athens, OH 45701 USA; 7 Division of Biomedical Engineering Sciences (BMES), Department of Basic Sciences, Loma Linda University, Loma Linda, CA 92354 USA; 8 Department of Biological Sciences, San Jose University, San Jose, CA 95192 USA

**Keywords:** Gene regulation in immune cells, Immunology, Biomarkers, Computational biology and bioinformatics, Gene regulatory networks

## Abstract

Using a ground-based model to simulate spaceflight [21-days of single-housed, hindlimb unloading (HLU) combined with continuous low-dose gamma irradiation (LDR, total dose of 0.04 Gy)], an in-depth survey of the immune and hematological systems of mice at 7-days post-exposure was performed. Collected blood was profiled with a hematology analyzer and spleens were analyzed by whole transcriptome shotgun sequencing (RNA-sequencing). The results revealed negligible differences in immune differentials. However, hematological system analyses of whole blood indicated large disparities in red blood cell differentials and morphology, suggestive of anemia. Murine Reactome networks indicated majority of spleen cells displayed differentially expressed genes (DEG) involved in signal transduction, metabolism, cell cycle, chromatin organization, and DNA repair. Although immune differentials were not changed, DEG analysis of the spleen revealed expression profiles associated with inflammation and dysregulated immune function persist to 1-week post-simulated spaceflight. Additionally, specific regulation pathways associated with human blood disease gene orthologs, such as blood pressure regulation, transforming growth factor-β receptor signaling, and B cell differentiation were noted. Collectively, this study revealed differential immune and hematological outcomes 1-week post-simulated spaceflight conditions, suggesting recovery from spaceflight is an unremitting process.

## Introduction

While the majority of spaceflight studies focus on the physiological responses during or immediately after flight, readaptation to nominal gravitational environments is also an important factor in astronaut health. This is particularly important for future exploratory missions to the Moon (0.166 g) or Mars (0.38 g) involving long-duration exposures to microgravity (0 g) and elevated doses of ionizing irradiation. Both the adaptation to spaceflight, and the readaptation to planetary gravitational environments, involve multiple physiological systems. Currently, short and the long-term consequences of prolonged exposure to deep spaceflight environments are largely unknown.

There are several consequences to living in low-Earth orbit. These include edema due to fluid and cardiovascular deconditioning^1^, myelin degeneration^2^, disorientation and motor control deficits^3,4^, visual deficits^5^, endocrine and hormonal alterations^6^, muscle tone wasting^7^, and bone loss due to lack of mechanical loading^8^. Upon return to Earth, there is a period of readaptation that includes acute motion sickness, unsteadiness, imbalance due to gravity-induced vestibular fluid shifts^9^, and blood pressure changes^10–12^. Most notably, space anemia^13,14^ was re-classified as post-flight anemia, because of inconsistencies reported in key hematological parameters, including red blood cell (RBC) counts and mean corpuscular volumes (MPV). This was attributed to the timing of blood sample collections occurring at post-flight return versus in-flight retrieval^10,14,15^. Mission duration can also affect post-flight recovery, whereby longer missions result in prolonged recovery post-mission^16^. This becomes an important risk factor to consider for exploratory missions to Mars. Indeed, understanding how immune and hematological cells compensate in response to the changing gravitational environment becomes particularly important as crew currently rehabilitate to Earth’s gravity for multiple months post-flight^17^. Yet, this timeframe may be problematic on planetary bodies with reduced gravity. Therefore, a better understanding of blood differentials and their transcriptional regulatory patterns are essential for successful development of countermeasures to be used on future exploratory missions to the lunar surface and Mars.

Ground-based models of spaceflight are developed to easily explore the impacts of the spaceflight environment on physiology. Indeed, we previously reported that a 21-day, protracted exposure of 0.04 Gy low dose/low dose rate gamma radiation (LDR), combined with hindlimb unloading (HLU), can have neurological impacts on blood–brain barrier integrity, the oxidative stress response, and neuroplasticity^18–22^. Here, we expand on this work, using RNA-sequencing of splenic tissues and complete blood cell (CBC) counts to characterize relevant immune and hematological pathways engaged after 1-week readaptation following simulated spaceflight conditions.

## Results

### Seven-days post-simulated spaceflight conditions result in distinct gene expression profiles

**Figure 1:**
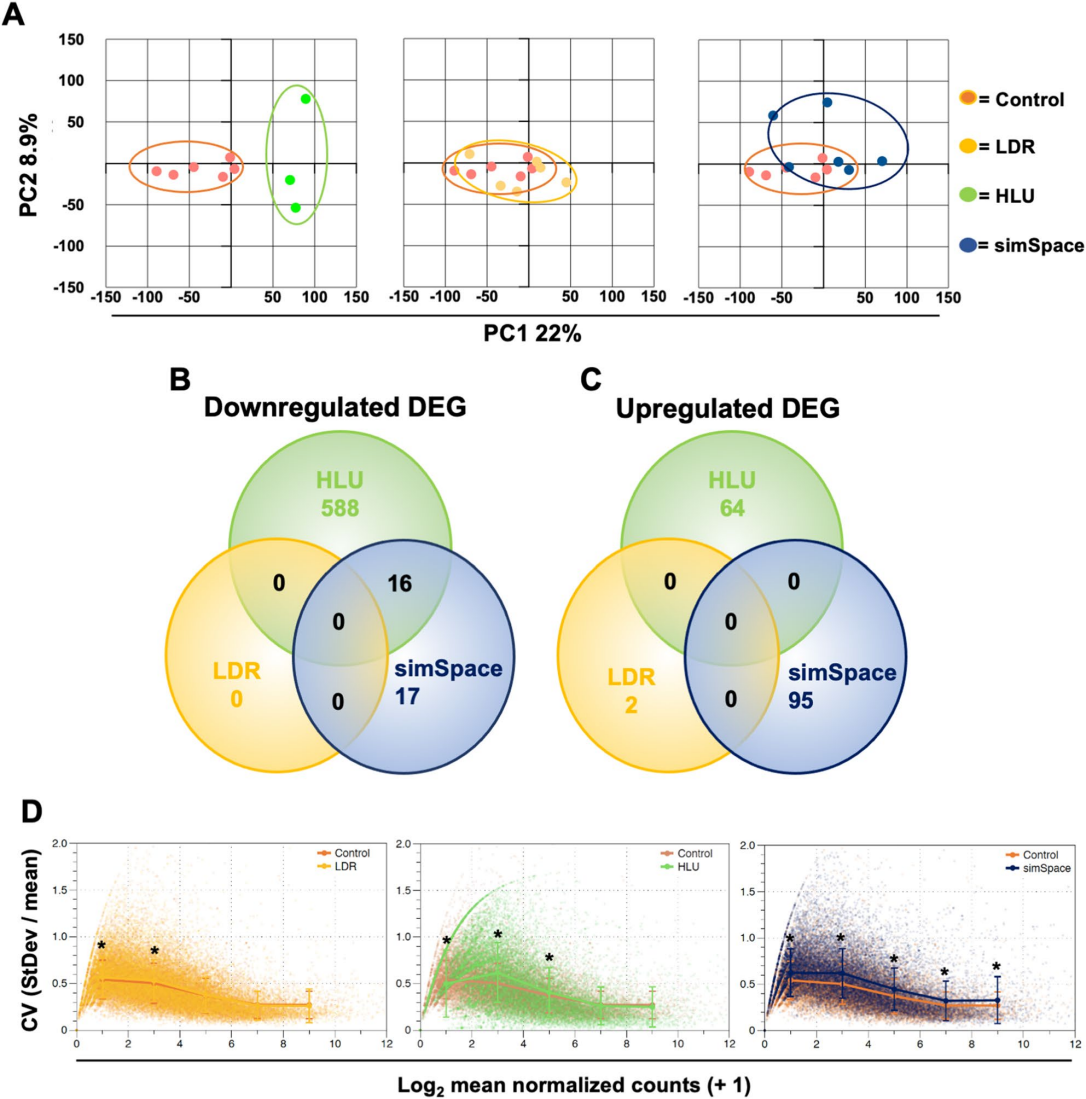
Post-simulated spaceflight conditions display distinct transcriptomics. Mice spleens collected 7-days following hindlimb unloading (HLU, green), low-dose irradiation (LDR, 0.04 Gy total dose, yellow), and combined HLU and LDR (simSpace, blue) for 21-days, compared to controls (orange). Principal component analysis (PCA) plot indicated for each condition (**A**). Venn diagrams for downregulated (**B**) and upregulated (**C**) genes. (**D**) Calculated coefficient of gene expression variation (CV) per gene plotted against the Log_2_ of the mean normalized count for that gene (+ 1) in each group. A one-way ANOVA with post hoc pairwise *t* tests (using step-down method, Sidak for adjusting p values) was performed in (**D**). Data represents n = 3–6 per group.

Principal component analysis (PCA) plots were generated with each cohort (Fig. 1A). Venn diagrams revealed no overlap of downregulated (Fig. 1B) nor upregulated (Fig. 1C) DEG. However, 33 genes were downregulated and 95 genes were upregulated in simulated spaceflight (simSpace) conditions combining LDR and HLU at 7-days post-exposure (Fig. 1B,C). In addition, the calculated coefficient of gene expression variation was determined for each experimental condition, which identified elevated variance in simSpace conditions (Fig. 1D).

**Figure 2:**
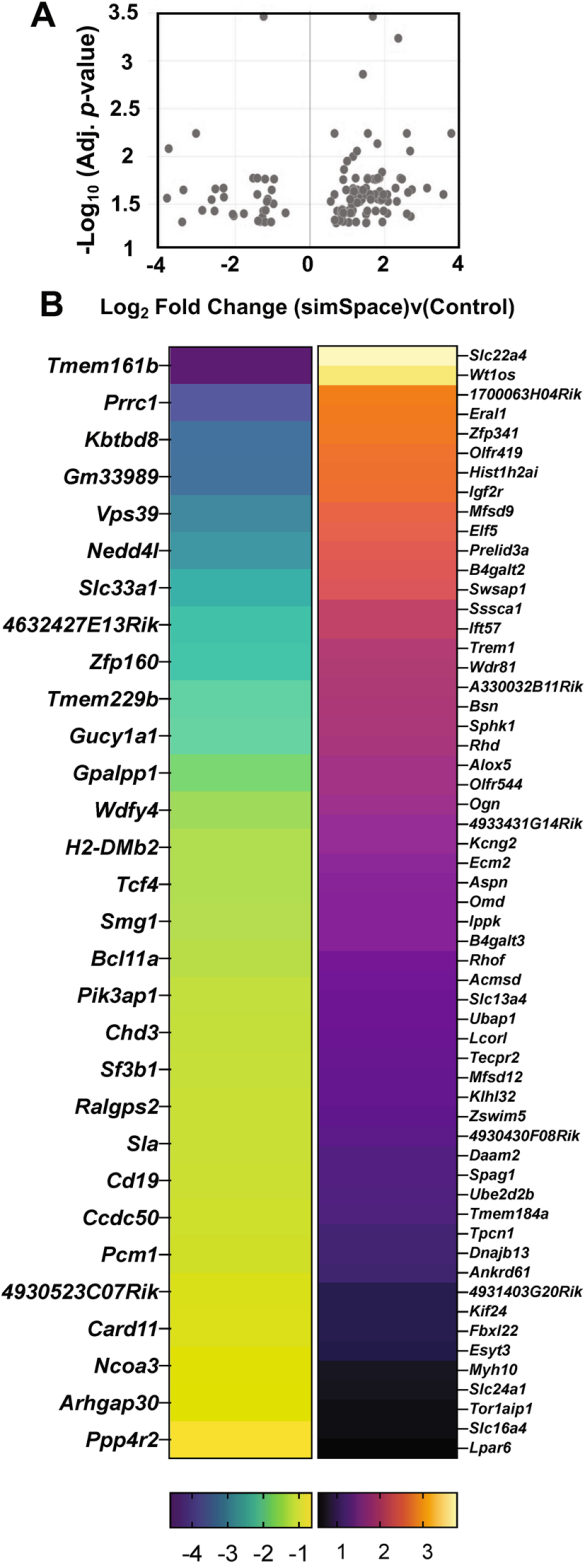
Differentially expressed genes profile following 7-days post-simulated spaceflight. (**A**) Volcano plot displaying Log_2_ fold change cutoff (0.263) compared to -Log_10_ (adjusted *p*-value, 0.05) adapted from GeneLabs visualization tool^23^ of simSpace versus controls. (**B**) Modified heatmap of downregulated (left panel) and upregulated (right panel) differentially expressed genes of simSpace versus controls (scale bar below panel). Data represents n = 6 per group.

As a secondary lymphoid organ, the spleen stores RNA-containing whole blood cells including white blood cells (WBC) or leukocytes, reticulated platelet and reticulocyte RBC, acting as a filter for circulating blood cells. Using NASA GeneLab’s volcano plot visualization tool with a maximum adjusted *p*-value of 0.05, statistically significant Log_2_ fold change of DEG (simSpace versus control) were plotted^23^ (Fig. 2A). Downregulated and upregulated genes associated with whole blood cell types include, *CD19*, *H2-DMb2*, *Alox5*, and *RhD* (Fig. 2B). The proportion of upregulated : downregulated DEG is approximately three : one (Fig. 2B). Collectively, 7-days post-simulated spaceflight conditions resulted in dysregulated immune and hematological system.

### Seven-days post-simulated spaceflight conditions reveal multiple Reactome pathway involvement

**Figure 3:**
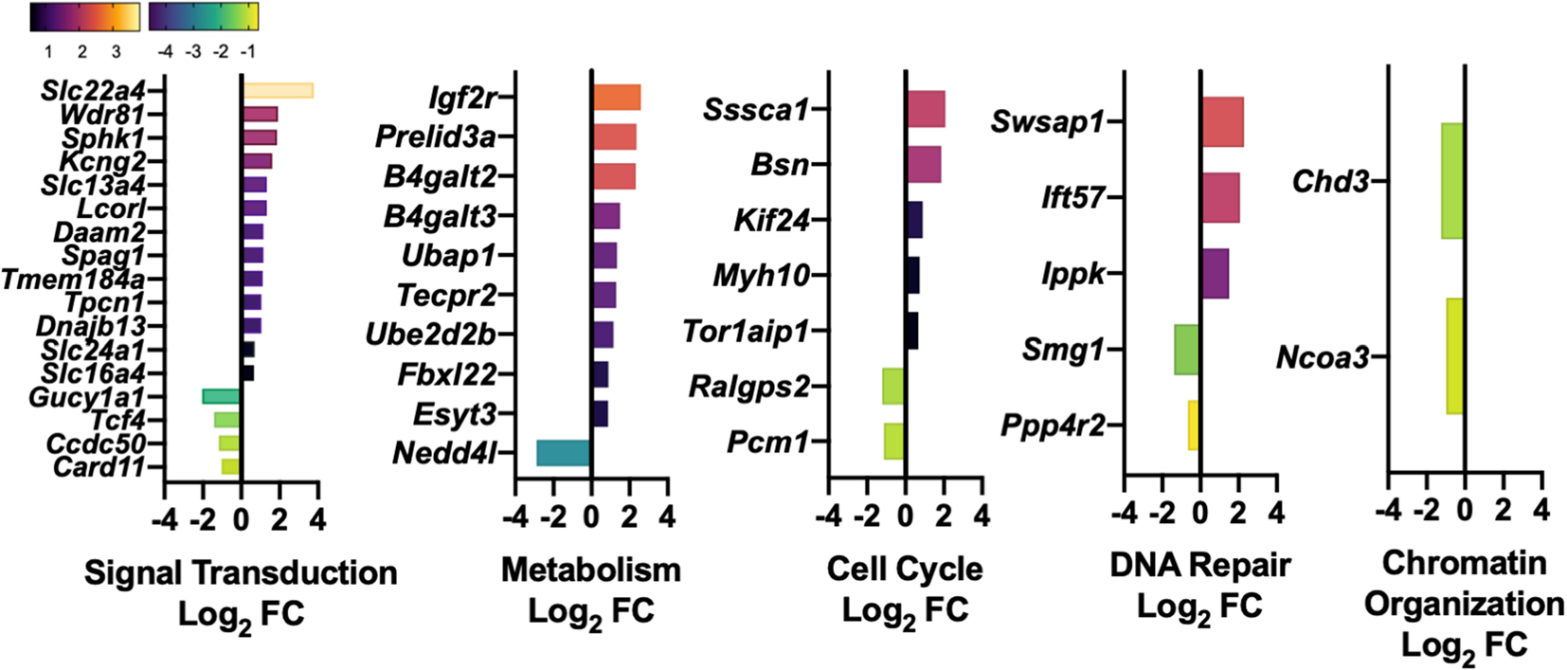
Post-simulated spaceflight revealed Reactome pathways involved in signal transduction, metabolism, cell cycle, chromatin organization, and DNA repair. Abridged Reactome pathway analyses of enriched DEG. Bar graphs of significant up (yellow) and down (purple) DEG (Log_2_ fold change, FC) (*p* > 0.05). Reactome pathways include, signal transduction, metabolism, cell cycle, DNA repair, and chromatin organization. Data represents n = 6 per group.

Murine Reactome pathway analysis was performed, which displayed pathways involved in signal transduction, metabolism, cell cycle, DNA repair, and chromatin organization at 7-days post-simSpace exposure (Fig. 3). The majority of DEG fall within the signal transduction pathway including multiple solute carrier family, *Slc* genes involved in transporter signaling of sodium/potassium and cation elimination. Metabolism-related genes involved in liposome and glycoprotein formation were also highly upregulated, including *Igf2r*, *B4galt2*, and *B4galt3* and the ubiquitin-pathways, including *Ubap1*, *Fbxl22*, and *Ube2d2b*. Cell cycle genes were also induced, including DEG associated with mitosis, such as *Sssca1* and *Kif24* and cytokinesis, such as *Myh10* and *Tor1aip1*. Induction of DNA damage repair genes were also noted including, recombination repair genes, *Swsap1* and *Ippk* and downregulation of DNA double stranded break repair gene, *Ppp4r2*.

### Marginal immune modifications at 7-days post-simulated spaceflight

**Figure 4:**
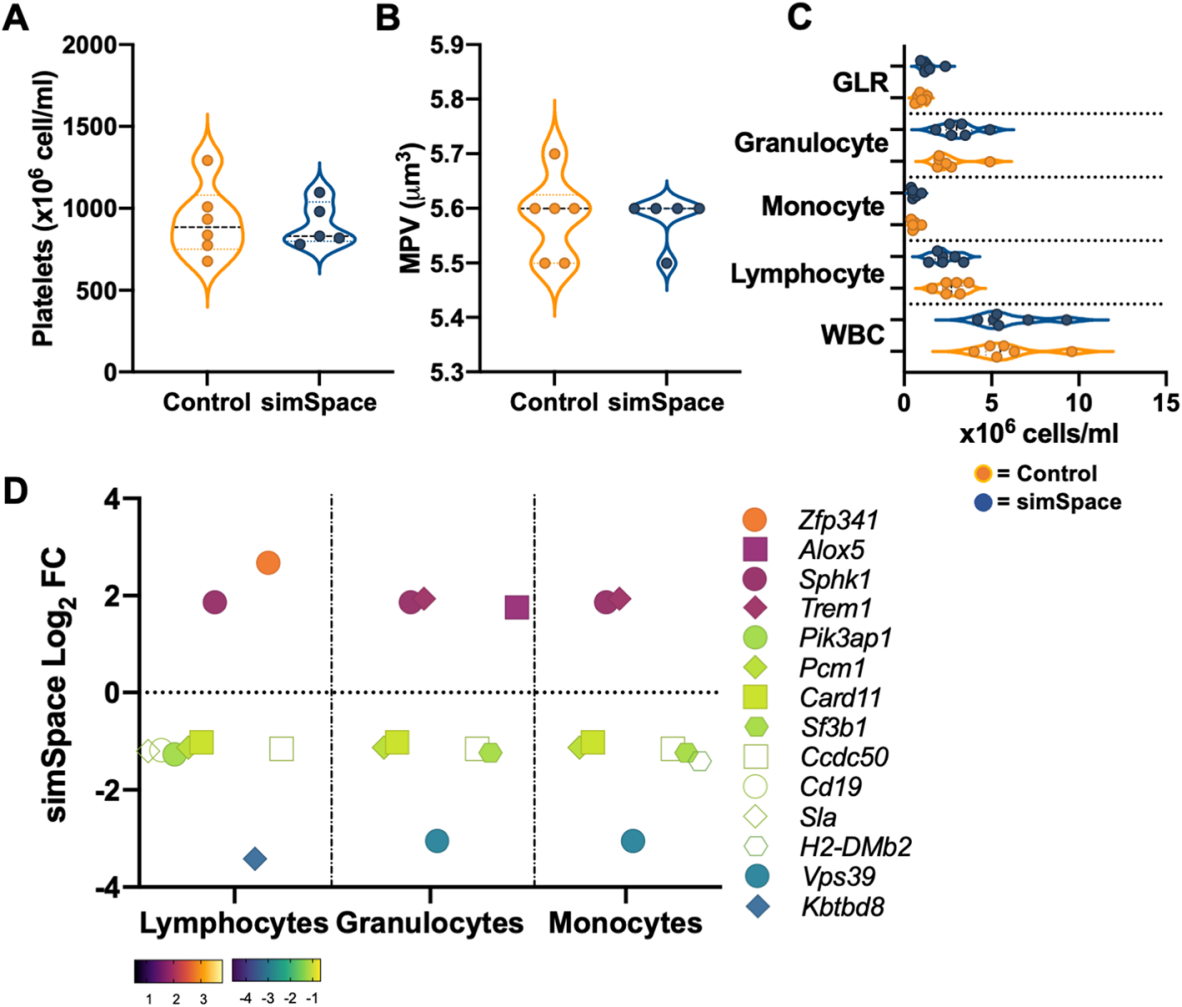
Splenic DEGs are altered post-simulated spaceflight, with minimal differences in immune population distributions in circulating blood. Whole blood was collected and platelets counts, × 10^3^ per mm^3^ (**A**) and MPV, mean platelet volume per cell, femtolitre (fL) (**B**) are displayed. (**C**) White blood cells (WBC), lymphocytes, monocytes, granulocytes, and granulocyte-to-lymphocyte ratio (GLR) are displayed for controls (orange) and simSpace (blue) conditions. (**D**) Significant upregulated and downregulated DEG (Log_2_ fold change) are displayed for each WBC subtype. Cell numbers were normalized to controls. Data represents ± SEM, n = 6 per group. An unpaired, parametric *t* test with Welch’s correction was performed for (**A**–**C**), *p < 0.05.

Blood was collected 7-days post-simulated spaceflight and WBC were counted with an automated analyzer, which displayed no significant differences between control and simSpace for immune differentials including, platelets (Fig. 4A), mean platelet volume (MPV, Fig. 4B), WBC, lymphocytes, monocytes, granulocytes, and granulocyte-to-lymphocyte ratio (GLR, Fig. 4C). Spleen DEG were categorized to specific leukocyte subtypes (monocyte, granulocyte, or lymphocyte) by their known functional output (Fig. 4D). Collectively, the results showed although there were no significant differences in population distributions, there were significantly different DEG between controls and post-simSpace cohorts with functional associations to leukocyte subtype.

### Alterations in hematological system at 7-days post-simulated spaceflight conditions

**Figure 5:**
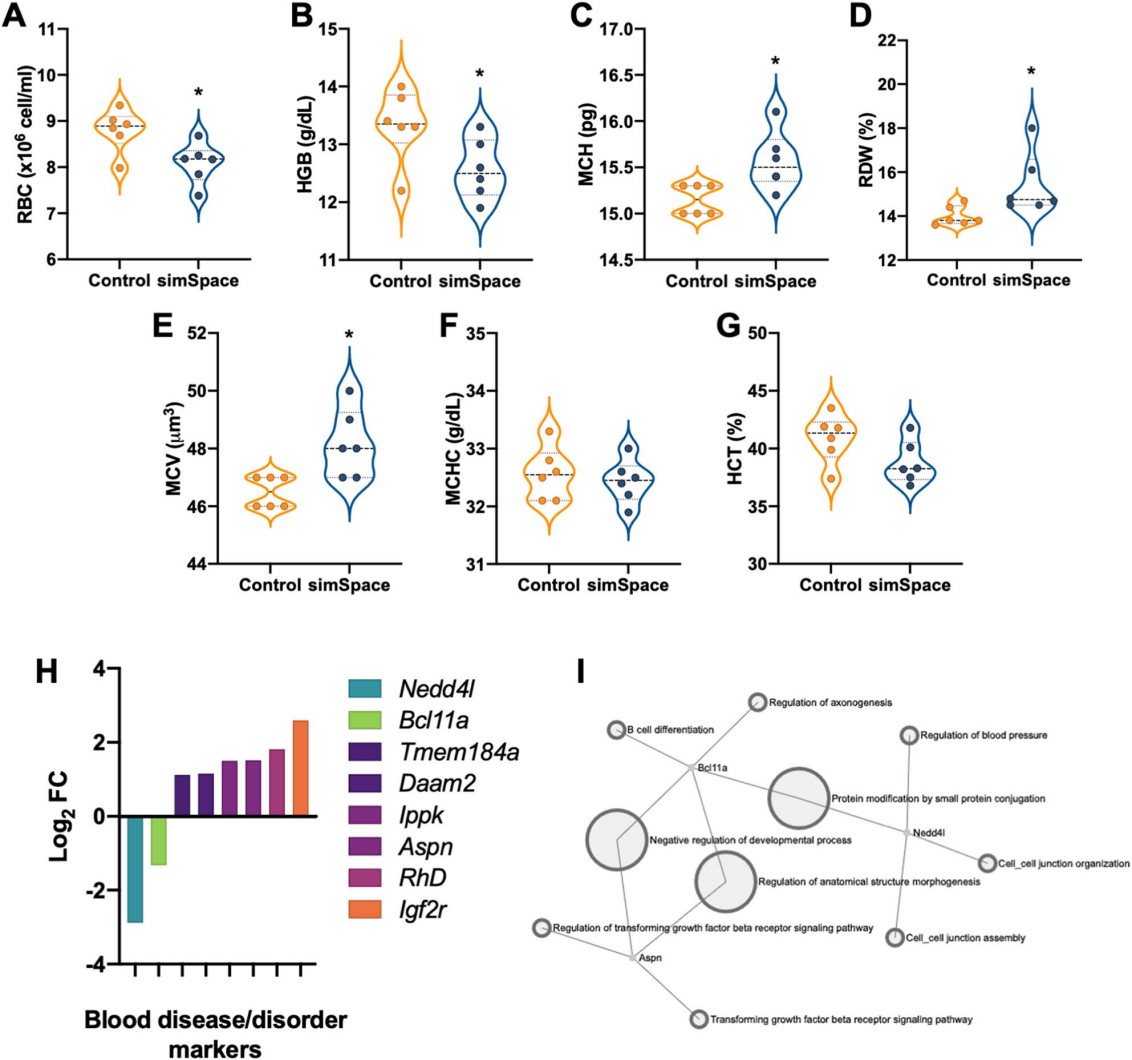
Distinct hematological gene expressions in spleens and red blood cell distributions in circulating blood post-simulated spaceflight. Whole blood was collected and red blood cell, RBC, × 10^6^ per mm^3^ (**A**), hemoglobin, HGB, g/dL (**B**), mean corpuscular hemoglobin, MCH, pg (**C**), mean corpuscular hemoglobin concentration, MCHC, g/dL (**D**), hematocrit, HCT, % (**E**), RBC distribution width, RDW, % (**F**), and mean corpuscular volume, MCV, fL (**G**). (**H**) Human blood disease/disorders as per Malacards Human Disease Databases (cite) displays human orthologs to mouse DEG. (**I**) Network Analyst Global EnrichNetwork tool mapped blood disease/disorder genes that shared common pathways. Cell numbers were normalized to controls. Data represents ± SEM, n = 6 per group. An unpaired, parametric *t* test with Welch’s correction was performed for (**A**–**G**), *p < 0.05.

CBC differentials were characterized by an automated hematological analyzer between control and post-simSpace cohorts. The results displayed significant reductions in RBC and hemoglobin (HGB) in post-simSpace cohorts, compared to controls (Fig. 5A,B). Significant increases in mean corpuscular hemoglobin (MCH), RBC distribution width (RDW), and MCV levels were revealed (Fig. 5C–E). No differences were noted between mean corpuscular hemoglobin concentration (MCHC) and hematocrit (HCT) levels (Fig. 5F,G). Murine spleen DEG that were orthologous to human blood disease/disorders markers, as defined by GeneCards Human Gene Database and MalaCards Human Disease Databases, were identified (Fig. 5H). Using the Network Analyst Global EnrichNetwork tool, blood disease/disorder markers were mapped to determine common pathways. The results showed three genes *Bcl11a*, *Nedd4l*, and *Aspn* had common pathways involved in regulation of blood pressure, protein modifications, transforming growth factor-β signaling, anatomical structure regulation, and negative regulation of developmental processes (Fig. 5I).

## Discussion

Recovery post-spaceflight is systemically taxing and often leaves a lasting impression on human health. Circulating RBC and WBC interact with all physiological systems; therefore, a systems biology approach was applied to assess the effects of the immune and hematological outcomes post-simulated spaceflight (simSpace). Since astronaut collections are difficult to acquire, ground-based animal models are a useful alternative that provides valuable insight into these outcomes. Therefore, spleens and blood of mice were analyzed by RNAseq and hematological analyses, respectively, at 7-days post-simSpace. Previous studies in our lab have developed a simSpace model of low-dose radiation (LDR, 0.04 Gy) and hindlimb unloading (HLU) that mimics astronauts in-orbit on the International Space Station (ISS)^18^. Analyses at 7-days post-simSpace provide insight into the immediate early responses similarly experienced by crew during Earth readaptation, a critical time point of equalizing homeostasis. Hematological profiling revealed minimal disparities in WBC differentials, however RBC displayed a wide variation in morphology and disrupted function, including reduced overall numbers and hemoglobin levels, suggestive of anemia. Transcriptomic analysis of the spleen, which is the filter for circulating blood cells and a secondary lymphoid organ, displayed genes involved in signal transduction, metabolism, cell cycle, chromatin organization and DNA repair pathways are altered. These analyses also revealed pathways connected to dysfunctional immunity and inflammation, including *Cd19, Alox5, Trem1,* and *H2-DMb2* expression. Genes also implicated in blood disorders, including *Bcl11a*, *Nedd4l*, and *Aspn*, with common pathways involved in regulation of blood pressure, protein modifications, transforming growth factor regulation, and negative regulation of developmental pathways were identified. Thus, understanding immediate early immune and hematological pathways engaged post-simulated spaceflight sheds light on suitable timepoints for therapeutic intervention to promote homeostasis and restoration.

DEG from spleens collected at 7-days post-simSpace were compared to single exposures of post-LDR or -HLU alone, which showed negligible overlap in genes (Fig. 1A–C), indicating distinct transcriptomic profiles are generated following each condition. Similar outcomes were also observed in our previous report that analyzed brain tissues at 4-months post-exposure^18^, indicating LDR, HLU, and simSpace independently trigger divergent pathways post-exposure. Interestingly, post-HLU appears to produce a large number of transcriptional changes compared to post-LDR alone, indicating altered gravity may cause pronounced physiological impairments. However, different doses and types of irradiation experienced on deep space missions would also cause distinct results^24^; therefore, future studies are aimed to delineate these transcriptional profiles as well. In addition, combined exposures of post-simSpace (HLU + LDR) compared to single exposures of post-HLU or post-LDR alone also created increased expression profile variability, which may account for reduced DEG observed in post-simSpace (Fig. 1D). A complete list of DEG for each condition is provided in supplemental information. We next wanted to determine DEG induced post-simSpace and found 33 genes were downregulated and 95 genes were upregulated, compared to controls (Fig. 2). Of note, no significant differences in overall body^18^ or spleen tissue weights (data not shown) were observed, supporting either minimal physiological effects occurred during simSpace or recovery post-simSpace has reached homeostasis.

Reactome analysis identified five major functional pathways that were affected, including metabolism, cell cycle, chromatin organization, DNA repair, and signal transduction (independent pathways from metabolism, cell cycle, chromatin organization, and DNA repair) (Fig. 3). The majority of DEG were grouped as signal transduction genes, including a large fold upregulation of the cation transporter, *Slc22a4*. In fact, multiple *Slc* genes were upregulated post-simSpace, which are transporters of small molecules, including sodium/potassium and cation elimination, suggesting systemic electrolyte imbalance and potential dehydration. Of note, these ion channels are also mechanosensitive and regulate signaling pathways via physical forces they receive^25^. Therefore, more studies are required to better understand the contribution of these mechanosensitive transporter signaling pathways during- and post-simSpace.

We and others have identified the oxidative stress response as a major pathway engaged during spaceflight^26–28^, while metabolic syndromes and ageing are also linked to oxidative stress^29^. In this study, multiple genes involved in metabolism were upregulated, including the insulin signaling receptor, *Igf2r*. Additionally, glycoprotein forming genes, *B4galt2* and *B4galt3* were both upregulated, suggesting heightened glycoprotein synthesis. Cell cycle pathways were also altered, including upregulation of genes *Sccca1, Kif24, Myh10,* and *Tor1aip1*. These DEG are involved in mitosis and meiosis via microtubule formation and cytokinesis, suggesting increased cellular turnover and division post-simSpace. Chromatin organization and remodeling genes *Chd3* and *Ncoa3* downregulation were also identified. Furthermore, *Swsap1, Ift57,* and *Ippk* genes were upregulated, indicating DNA repair mechanisms occur post-simSpace. Collectively, multiple DEG pathways were actively engaged at 7-days post-simSpace, indicating a dynamic and constant healing process seeking homeostasis.

Immune-related genes were grouped according to their cellular function. Myeloid cell (monocyte and granulocyte)-related genes included downregulation of *Card11, Sf3b1*, *H2-DMb2, Vps39, Ccdc50*, and *Pcm1*; while *SphK1*, *Trem1*, and *Alox5* were upregulated (Fig. 4D). *H2-DMb2* is part of the MHC complex involved in antigen presentation to lymphocytes^30^. Downregulation of this gene would imply impaired antigen presentation and deficient immunological activation; however, this may be an important mechanism post-simSpace to dull the immune response, as recovery should be devoid of self-antigenic stimulation. Furthermore, downregulation of *Vps39* (involved in fusion of endosomes/lysosomes) suggests phagocytosis impairment^31^. In line with this, impairment of neutrophil phagocytosis is observed up to 3-days post-spaceflight^32,33^, suggesting a possible role for downregulated *Vps39* in this process. The transcription factor NFκB activates a wide variety of signaling pathways involved in immune activation, inflammation, and cytokine production^34^. *Card11* and *Ccdc50* are both regulators of NFκB signaling. Their expression levels are downregulated, indicating dysregulated immunity. In addition, *Alox5* is typically upregulated by the proinflammatory cytokine, GM-CSF, involved in the generation of emergency myelopoiesis and activates matured myeloid cells into a pro-inflammatory state^35^. Therefore, *Alox5* upregulation suggests a proinflammatory environment predominates post-simSpace. In line with this, *Trem1* is upregulated and is involved in amplifying inflammation of neutrophils and monocytes^36^. Indeed, *Trem1* inhibition has been successfully shown to prevent *mir-155*-driven lung inflammation and injury^37^, septic shock^38^, and neuroinflammation^39^. *Trem1* is expressed in neutrophils, which are granulocytes^40^. Elevated granulocytes and granulocyte-to-lymphocyte ratio (GLR) has been identified as a biomarker for inflight immune monitoring^26,41^, therefore, it is plausible that *Trem1* induction may also be a determinant for inflight inflammation. Acute inflammation is beneficial in the face of immunological challenges, such as infections or tumors, yet chronic persistence, specifically post-exposures can be detrimental to the host. Resolution of inflammation does not appear to occur at 7-days post-simSpace, suggesting the potential disease development^42^. Furthermore, although correlation of *Alox5* and *Trem1* expression levels and severity of inflammation is not clear, monitoring *Alox5* and *Trem1* upregulation may be useful biomarkers to measure chronic inflammation in future spaceflight studies.

Lymphocytes play an important role in cell-mediated and humoral immunity. Notable DEG altered post-simSpace, included downregulation of *Sla, Cd19, Pikap1, Kbtbd8, Pcm1, Card11*, and *Cccdc50;* and upregulation of *Sphk1* and *Zfp341* (Fig. 4D). Of note, *Sla* negatively regulates TcR signaling, which may contribute to issues during positive selection and non-specific antigen-immunity^43^. Indeed, T cell function is impaired during- and post-spaceflight^44,45^, therefore supporting identification of gene pathways involved in this process for future studies. Additionally, *Cd19* and *Pik3ap1,* are involved in B cell phenotypes, including development and BcR signaling^46,47^, therefore downregulation suggests deficiency in humoral immunity. Certainly, B cells are impacted by ground-based deep space simulation models^24^ and post-spaceflight^48^. Further, activation of splenocytes collected 3-days post-flight produced a robust elevation of the inflammatory chemokine MIP-1α, further supporting a proinflammatory state post-spaceflight^44^. Yet, the overall contribution of immune responses on whole-organism physiology cannot be fully concluded until longitudinal phenotypic studies are performed, as mission timeframes vary, ground-model conditions and exposure timeframes are non-standardized, and post-mission/model tissue collections differ, which can all result in different interpretations. Nonetheless, altered immunity is recognized across multiple in-flight, at readaptation, and in ground-based models of spaceflight, suggesting a need to delineate all potential mechanisms involved, and strongly supports the need for personalized medicine for future crewmembers.

CBC indicated no differences in WBC counts, which may be due to either group-housing effects on cell distribution^49^ or homeostasis is established at this time point post-simSpace. There were, however, significant reductions in overall RBC numbers and HGB levels (Fig. 5A,B). These results suggest at 7-days post-simSpace anemic conditions persist, which may explain post-flight etiology during return to Earth’s gravity in crewmembers^9–11^. Other parameters showed no significant differences including, MCHC and HCT (Fig. 5D,E). However, significantly increased levels of mean corpuscular hemoglobin (MCH), RBC distribution width (RDW), and MCV (Fig. 5C,F,G). Collectively, abnormal RBC shape suggest anemic outcomes occurred post-simSpace. Furthermore, both elevated or reduced values of MCH, RDW, and MCV indicate abnormal size and volume of RBC, which impacts hemoglobin function and oxygen carrying capacity, contributing to anemic states^50,51^. An elevated RDW can also contribute to cardiovascular and cancer risk^52^, highlighting the necessity to normalize these biomarkers during post-flight readaptation. Interestingly, elevated RDW may be caused by elevated inflammation^53^, which is also observed in our model, therefore targeting inflammation early post-flight may avert cardiovascular- or cancer-related risks. In line with this, RDW was the only hematological marker that remained elevated at 30-days post-simSpace, while reaching homeostasis at 270-days post-simSpace (data not shown), suggesting the importance of regulating this biomarker in particular for future spaceflight studies.

Since RBC do not contain a nucleus and thus genetic material, the majority of blood disease/disorder genes are likely pooled from DNA-containing immature RBC or reticulocytes, immature platelets or reticulated platelets, WBC, and other cells of the spleen, including mesothelial cells and smooth muscle cells^54^. Using Malacards Human Disease Database, outstanding blood disease/disorder orthologs including, upregulated *Tmem184a, Daam2, Ippk, Aspn, RhD*, and *Igf2r;* and downregulated *Nedd4l* and *Bcl11a* were identified (Fig. 5H). For one, an elevated *RhD* (Rh factor antigen) was noted post-simSpace, which may have been induced due to RBC loss, as Rh(null) phenotypes are associated with hemolytic anemia^55^. As it is unclear if anemia was induced during- or post-simSpace in our model, and due to the complex definition of space/post-flight anemia, more studies are required. Nonetheless, while determining the timeframe of anemia onset is important, we propose the potential for prolonged and pronounced anemia manifestation in astronauts on partial gravity surfaces of the Moon and Mars, following long duration, deep space travel. Therefore, identifying biomarkers to assist with future countermeasure developments to help safeguard astronauts on future deep space missions is critical. Interestingly, pathway analysis implicated *Bcl11a, Aspn*, and *Nedd4l* are interconnected and regulate a wide variety of pathways including, blood pressure, B cell differentiation, and TGF-βR signaling (Fig. 5I). Due to fluidic shifting and reduced normal loading in spaceflight, astronauts returned to Earth are at an increased risk of low blood pressure when standing^12^ and arterial stiffening^56^. Therefore, targeting these interconnected genes may provide a robust countermeasure suited to reverse both immune and cardiovascular disparities during readaptation. We recognize complex pathways depend on multiple genes for function. Therefore, differential gene expression of a few may not disrupt biological phenotypes, and as such additional studies are required to assess functional consequences. Furthermore, physiological responses depend on multiple gene expressions within collective pathways; therefore, a thorough survey of the pan-transcriptomic landscape is required to construct reliable countermeasures.

In brief, this study identified key DEG and DEG pathways engaged in systemic blood circulation via spleen transcriptome and CBC differential analyses at 7-days post-simSpace. Primary responses include persistent inflammation and anemia are protracted up to 7-days post-simSpace. Limitations of this study include restricted cross-translation to humans, lack of baseline and longitudinal results, and absence of galactic cosmic ray radiation testing parameters. Nonetheless, future space biology queries can be generated from this study. Furthermore, this study highlights the requisite of systemic homeostasis upon return to Earth’s gravity and describes options for recourse during reduced gravity experiences. In brief, comprehensive and dynamic outcomes of the immune and hematological systems occur 7-days post-protracted simulated spaceflight.

## Methods

### Experimental conditions

Six-month-old, female *C57BL/6J* mice (Jackson Laboratory) were acclimatized in standard habitats at 20 °C with a 12 h:12 h light:dark cycle for 7 days. Following acclimatization, animals were housed one per cage and assigned to one of four groups: (1) control (*n* = 6); (2) hindlimb unloaded (HLU) (*n* = 3); (3) low-dose irradiated (LDR, total dose 0.04 Gy) (*n* = 6); and (4) simSpace, combination of hindlimb unloaded and low-dose irradiated (HLU + LDR) (*n* = 6) for 21 days. Low-dose/low-dose rate was delivered using ^57^Co plates (a total dose of 0.04 Gy at 0.01 cGy/h) placed 7 cm below the cages, with 1 plate per 2 cages for whole-body irradiation. Uniformity of dose was ± 5%, as previously described^18–22^. Post-exposure animals were group housed (3 per cage) for 7-days. Commercial pellet chow (5LG4, LabDiet^R^) and hydrogel were available ad libitum*.* Health status, water and food intake were daily monitored. After 7-day post-simSpace, mice were CO_2_ euthanized and spleens were collected and frozen. RNA was isolated from whole, frozen spleens and samples were subjected to whole transcriptome shotgun sequencing (RNA-sequencing)^18^. Datasets were deposited to the NASA GeneLab Data Systems (GLDS)-211 and were analyzed using the NASA pipeline for determination of differentially expressed genes (DEG)^23^. All methods were performed in accordance with the guidelines recommended in the Guide for the Care and Use of Laboratory Animals^57^ and was approved by the Institutional Animal Care and Use Committee (IACUC) at Loma Linda University (Protocol number 8130028). This study is reported in accordance with ARRIVE guidelines^58^.

### Complete blood count differential analysis

Mice were euthanized with 100% CO_2_ at day seven after the simulated spaceflight period of 21-days. Whole blood was collected via cardiac punch in [K2]-ethylenediaminetetraacetic acid (EDTA) coated syringes immediately following euthanasia and evaluated using the ABC Vet Hematology Analyzer (Heska Corp., Waukesha, WI, USA). Parameters characterized include red blood cell (RBC), platelet (PLT), and white blood cell (WBC) counts, hemoglobin concentration (HGB) and hematocrit (HCT, percentage of whole blood consisting of RBC). Mean corpuscular volume (MCV, mean volume per RBC), mean corpuscular hemoglobin (MCH; mean weight of hemoglobin per RBC), mean corpuscular hemoglobin concentration (MCHC; concentration of hemoglobin per RBC), RBC distribution width (RDW; width of the RBC histogram produced by cell number x cell size), and the mean platelet volume (MPV; volume per cell) were also reported, along with absolute counts of granulocytes, monocytes, and lymphocytes (× 10^6^ cells/ml).

### Tissue collection and nucleic acid extraction

Mice were humanely CO_2_ euthanized followed by immediate exsanguination by cardiac puncture.

Spleens were isolated and placed in a sterile cryovial, snap frozen in liquid nitrogen and kept at − 80 °C. Spleens were homogenized with CKMix (Bertin Instruments, Montigny-le-Bretonneux, France) and a Minilys homogenizer (Bertin Instruments). AllPrep DNA/RNA/miRNA Universal Kit (Qiagen, Hilden, Germany) extracted RNA and DNA according to the manufacturer’s instructions. RNA and DNA concentrations were measured using a Qubit 3.0 Fluorometer (Thermo Fisher Scientific, Waltham, MA, USA) and stored at − 80 °C.

### RNA sequencing

To construct RNA-sequencing libraries, an Ovation Mouse RNA-Seq System 1–96 (NuGEN Technologies, Redwood City, CA, USA) was used per manufacturer’s instructions. RNA integrity was determined using a 2100 Bioanalyzer (Agilent) with RIN values > 8. 100 ng of total RNA was used as input. cDNA (first and second strands) was synthesized from total RNA spiked with ERCC ExFold RNA Spike-In Mix 1 (Life Technologies, Carlsbad, CA, USA) at the appropriate ratio. Products were sheared using Covaris S220 Focused-ultrasonicator (Covaris Inc., Woburn, MA, USA) to obtain fragment sizes between 150–200 bp. Followed by end-repair, adaptor index ligation and strand selection. For multiplexing, barcodes with unique indices out of 96, were used per sample. Custom InDA-C primer mixture SS5 Version5 for mice (NuGEN Technologies) was used for strand selection. Libraries were amplified by PCR (17 cycles) on a Mastercycler Pro (Eppendorf) and purified with RNAClean XP Agencourt beads (Beckman Coulter, Pasadena, CA, USA). Libraries were sequenced on a HiSeq 4000 (Illumina, Mira Loma, CA, USA) to generate 15–30 M 75-bp single end reads per sample. Raw data are available at NASA GeneLab (genelab.nasa.gov, accession GLDS-211)^23^.

### Differential expression and pathway enrichment analysis

With Cutadapt^59^ using the Trim Galore! wrapper, sequencing reads were trimmed. All reads were mapped with STAR^60^ and expressions were quantified and imported with RSEM^61^ and tximport^62^ respectively, into DESeq2 (R Bioconductor)^63^. Log_2_ fold-change (0.263) and adjusted *p*-value cutoffs (0.05) were used. All groups were compared using the Wald test and the likelihood ratio test was used to generate the F statistic p-value. Reactome version 67^64^ was used to detect enriched murine pathways. Volcano plots were generated using NASA’s GeneLab tool^23^. Murine spleen DEG that were orthologous to human blood gene-related diseases, as defined by GeneCards Human Gene Database and MalaCards Human Disease Databases, were identified. Using the Network Analyst Global EnrichNetwork tool, with parameters including the gene ontology: biological process (GO:BP) enrichment analysis database, large graph layout, and single node display (https://www.networkanalyst.ca/NetworkAnalyst/Secure/vis/ListEnrichment.xhtml), the blood gene-related gene list was mapped to determine common pathways.

### Statistical analysis

Differential expression analysis was performed in R DESeq2. All groups were compared using the Wald test and the likelihood ratio test was used to generate the F statistic p-value. A one-way ANOVA with post-hoc (Sidak) test was performed for the calculated coefficient of gene expression variation per gene. An unpaired, parametric *t* test with Welch’s correction was performed for all CBC parameters. A GitHub data analysis notebook was created and can be found here, https://github.com/jgalazka/GLDS-211_Paul_2021.

## Supplementary Information

Supplementary Information 1.

Supplementary Information 2.
