# Supplementary material for: Immunological and hematological outcomes following protracted low dose/low dose rate ionizing radiation and simulated microgravity
Source: Sci Rep. 2021 Jun 1;11:11452. doi: 10.1038/s41598-021-90439-5 (PMC8169688; doi:10.1038/s41598-021-90439-5)
Supplement: Supplementary file 2 — Supplementary Information 2. [file 41598_2021_90439_MOESM2_ESM.docx]

**Supplementary Information**

**Supplementary Table 1.** Differentially expressed genes (DEG, Log_2_ fold change cutoff (0.263) and adjusted *p-value* < 0.05) from splenic tissue of mice seven-days following 21-days of protracted LDR (Total dose = 0.04 Gy), HLU, and simSpace (LDR and HLU). Data represents n = 3-6 per group. Up- and down-regulated genes are described in each condition.
